# Editorial: Challenges of COVID-19 in dermatology patients on immunosuppression: Risk, outcome, vaccination, and beyond

**DOI:** 10.3389/fmed.2022.987534

**Published:** 2022-08-09

**Authors:** Hamidreza Mahmoudi, Aikaterini Patsatsi, Dedee F. Murrell, Soheil Tavakolpour, Maryam Daneshpazhooh

**Affiliations:** ^1^Autoimmune Bullous Diseases Research Center, Department of Dermatology, Razi Hospital, Tehran University of Medical Sciences, Tehran, Iran; ^2^Faculty of Health Sciences, Aristotle University of Thessaloniki, Thessaloniki, Greece; ^3^Department of Dermatology, St George Hospital, University of New South Wales, Sydney, NSW, Australia; ^4^Department of Cancer Immunology and Virology, Dana-Farber Cancer Institute, Harvard Medical School, Boston, MA, United States

**Keywords:** COVID-19, SARS-CoV-2, psoriasis, melanoma, vaccination, autoimmune bullous diseases

Corticosteroids, other immunosuppressive drugs, and biological agents are key elements of the treatment of immune-mediated diseases. There is limited data available about the outcome of dermatology patients on immunosuppressants who are infected with SARS-CoV2. Meanwhile, the release of pro-inflammatory cytokines and the so-called cytokine storm of COVID-19 may trigger the onset or exacerbation of autoimmune or autoinflammatory diseases.

On the other hand, COVID 19 vaccines are considered the game-changer of the pandemic. Some issues related to SARS-CoV2 vaccination should be addressed in dermatology patients, most notably, vaccine efficacy in patients on immunosuppressants and the risk of worsening of autoimmune diseases.

One of the most intriguing questions is whether the cytokine storm and the immune system overreaction after COVID-19 infection may induce the development either of a new autoimmune disease or the relapse of an existing one. In this issue, Lotfi et al. describe a rare case of pansclerotic morphea that rapidly progressed a few weeks after infection with COVID-19 in a woman with no history of any autoimmune skin or rheumatic disease. Drenovska et al. also report a woman with preexisting chronic cutaneous lupus erythematosus controlled with topical corticosteroids and photoprotection. She developed a flare of disease as Rowell syndrome with erythema multiforme-like lesions and high anti-Ro and anti-Ro B2 antibodies 2 weeks after a SARS-CoV-2 infection. It is important to enrich the existing literature with similar cases and add knowledge about the outcomes of COVID-19 infection for further research.

Another intriguing concept is the impact of the COVID-19 vaccine on the clinical course of autoimmune diseases. In a single-center study from Taiwan, Huang and Tsai reported 15 episodes of psoriasis worsening and morphological changes in 51 patients with psoriasis likely due to Th17 activation after vaccination. Additionally, all but one of the patients who received two doses of vaccination experienced disease exacerbation after the first shot but not the second. Under the same concept, COVID-19 vaccines may induce bullous pemphigoid, as reported in an Italian multicenter study by Maronese et al. collected clinical, histopathological and immunopathological data of 21 patients with new onset bullous pemphigoid (BP) associated with COVID-19-vaccines.The authors concluded that, in this subset of patients, there are slight differences between BP possibly triggered by COVID-19-vaccines and classical BP, such as a male predominance and a reduced humoral response to BP230.

Many theories have been proposed regarding the pathogenic mechanisms of autoimmunity following viral infection or vaccination; one of them is molecular mimicry. Kasperkiewicz et al. examined this hypothesis by testing the sera of 12 seropositive post-COVID-19 individuals and 12 seropositive healthy volunteers who received two doses of an mRNA COVID-19 vaccine for autoantibodies to the main immunobullous autoantigens. Interestingly, none of the subjects had concomitant antibody reactivity. The authors concluded that their results argue against a relationship between SARS-CoV2 infection/vaccines and AIBDs with respect to disease-triggering antibody cross-reactivity.

During the pandemic, especially before vaccination, the potential benefits and risks of the use of immunosuppressants and biologics, especially rituximab in AIBDs, were under continuous discussion. Miyamoto et al. report a case series of four pemphigus patients from Brazil who required adjustment of treatment and present the challenges of therapeutic decisions. It is considered to be extremely important to monitor B-cell recovery after anti- CD 20 therapy, in order to determine the most appropriate timing to vaccinate patients and achieve a maximized seroconversion. The authors also suggest that additional studies are necessary to evaluate COVID-19 outcomes in vaccinated AIBD patients with the aim to better understand the safety of immunosuppressive and biologic treatments after immunization.

Biologic treatment is another hot topic in the COVID-19 era. In the review article prepared by Zeng et al., the authors discuss the pearls and pitfalls of using biologic treatments in patients with psoriasis during the COVID-19 pandemic. Although the exact consequences of the treatment on the risk of COVID-19 infection and severity have not been determined yet, the authors suggested that, according to the available data, there is a low risk of severe COVID-19 infection in patients being treated with anti-TNF-α, IL17, and IL23 inhibitors. Therefore, none of the biologic treatments mentioned in this article is likely to result in serious adverse effects for patients with COVID-19. Nonetheless, it is important to carefully assess the impact of such treatments during the pandemic.

Melanoma is the most lethal form of skin cancer, and the COVID-19 pandemic may have a profound impact on the diagnosis, treatment, and follow-up of patients suffering from melanoma. As part of their comprehensive review, Li et al. discussed practical points regarding screening, diagnosis, surgical treatment, and the use of new treatment options in patients with melanoma during the COVID-19 era.

As the COVID-19 era unfolds, there is increased concern regarding the effects of using immunosuppressive agents in the development of successful immunity to SARS-CoV-2 vaccines. Benucci et al. examined this hypothesis in 110 patients with psoriatic arthritis receiving immunomodulatory therapy (anti-TNF-a, anti-IL17, methotrexate). As compared with the control group, the selected patients demonstrated a reduced humoral response. Even though the antibody response did not differ significantly between groups treated with different medications. In another study on a small group of dermatological patients, Seree-aphinan et al. observed decreased humoral immune responses after a complete course of an inactivated vaccine in participants using azathioprine, cyclosporin, mycophenolate mofetil, or prednisolone >10 mg/day compared to those receiving methotrexate <10 mg/week, prednisolone <10 mg/day, or secukinumab, ixekizumab, or omalizumab. They concluded that poor responders may benefit from vaccine platforms that trigger a greater level of immunogenicity or booster doses.

Overall, the articles in this Research Topic highlight the challenges of dermatology patients in the COVID-19 era among them worsening of autoimmune diseases by SARS-CoV2 infection/vaccine, and reduced immunogenicity of vaccines and provide us with a clearer insight into the interaction between COVID-19 and skin disorders.

## Author contributions

Conceptualization: HM, AP, DM, ST, and MD. Data curation: HM, AP, and MD. Supervision and writing- review and editing: DM and MD. Writing-original draft: HM, AP, ST, and MD. All authors contributed to the article and approved the submitted version.

## Dedication

This issue is dedicated to the challenges dermatology patients have been facing during the COVID-19 pandemic.

## Conflict of interest

The authors declare that the research was conducted in the absence of any commercial or financial relationships that could be construed as a potential conflict of interest.

## Publisher's note

All claims expressed in this article are solely those of the authors and do not necessarily represent those of their affiliated organizations, or those of the publisher, the editors and the reviewers. Any product that may be evaluated in this article, or claim that may be made by its manufacturer, is not guaranteed or endorsed by the publisher.

